# Positive airway pressure delivery: overcoming old hurdles, exploring new frontiers

**DOI:** 10.3389/frsle.2024.1522635

**Published:** 2024-12-20

**Authors:** Ludovico Messineo, David P. White, William H. Noah

**Affiliations:** ^1^Division of Sleep and Circadian Medicine, Harvard Medical School, Brigham and Women's Hospital, Boston, MA, United States; ^2^Sleep Centers of Middle Tennessee, Murfreesboro, TN, United States

**Keywords:** inspiratory pressure, positive airway pressure, KairosPAP™ (KPAP™), V-Com^®^, PAP algorithms, pressure support

## Abstract

Despite being the gold-standard treatment for obstructive sleep apnea (OSA), continuous positive airway pressure (CPAP) faces important challenges, particularly with patient adherence. Many individuals find CPAP difficult to tolerate due to noise, social inconveniences, characteristics inherently linked to their sleep disorder and side effects, including mask discomfort, air leaks, nasal congestion, and the unnatural sensation of exhaling against positive pressure. All this often leads to reduced usage, limiting CPAP's potential to deliver long-term health benefits. This review revisits the dynamics of pharyngeal collapse during sleep on PAP, offering a new interpretation that challenges the long-standing view that higher inspiratory pressure is required to maintain pharyngeal patency. Emerging evidence, combined with the knowledge from older studies, suggests that airway collapse often occurs near end-expiration, which may be the only time that substantial positive airway pressure is required. Efforts to improve CPAP compliance have reduced expiratory pressure, leading to the introduction of bilevel PAP (BPAP) and expiratory pressure relief algorithms, which may cause airway destabilization, without yielding the improvements in adherence that were initially anticipated. Thus, despite over three decades of innovation, which have also seen heated humidifiers and tubes, customized 3D-printed masks and auto-titrating PAP come to market, there has been limited success in systematically increasing long-term CPAP adherence rates. In response, we discuss novel approaches such as $\dot{\text{V}}$-Com^®^ and KairosPAP™ (KPAP™), which reduce inspiratory pressure and, in the case of KPAP™, also much of expiratory pressure, returning to full pressure only at the end of expiration. Recent studies suggest these technologies improve comfort and reduce unintentional leaks and may lead to better adherence without sacrificing treatment effectiveness. This aligns with the hypothesis that stabilizing the airway during end-expiration may be key to enhancing CPAP comfort and adherence. In conclusion, while technological advancements have improved the CPAP experience, further progress will likely come from solutions that better address patient comfort with the applied pressure. KPAP™ is one such innovation with the potential to enhance adherence, but additional research is needed to fully understand its long-term impact and effectiveness in PAP therapy for OSA.

## 1 Introduction

Obstructive sleep apnea is characterized by chronic, self-propagating narrowing—or obstruction—of the upper airway. To date, more than 40 years after its discovery (Sullivan et al., 1981), continuous positive airway pressure (CPAP) remains the gold-standard treatment for OSA due to its high efficacy in resolving respiratory events. For example, CPAP, differently than other OSA treatments such as mandibular advancement devices or hypoglossal nerve stimulation, addresses OSA through mechanically splinting the upper airway open, regardless of the underlying cause of obstruction, e.g., impaired anatomy, low lung volume or increased arousability (Messineo et al., 2024a,c; Messineo and Eckert, 2022). In addition, substantial evidence supports CPAP's effectiveness in alleviating OSA-related symptoms (McDaid et al., 2009; Kuhn et al., 2017) and reducing blood pressure (Gottlieb et al., 2014). However, large, multi-centric randomized controlled trials have failed to show a clear protective effect of CPAP on cardiovascular outcome in OSA patients (Sanchez-de-la-Torre et al., 2020; Labarca et al., 2020; McEvoy et al., 2016; Peker et al., 2016). This may be dependent on an actual lack of a protective effect of CPAP on the cardiovascular system, but could also stem from several other factors: (1) the apnea hypopnea index (AHI) may be an inadequate biomarker for patient selection compared to more sensitive measures such as hypoxic burden (Messineo et al., 2024a), (2) participants in these RCTs may not accurately represent real-world OSA populations (Gerves-Pinquie et al., 2022), (3) CPAP pressures may lead to the release of pro-inflammatory mediators secondary to stretch of endothelial or epithelial cells in the lung, which could increase the risk of adverse cardiovascular events (Peker et al., 2024; Gottlieb et al., 2022; Shah et al., 2023), and (4) poor adherence among trial participants may skew results (Sanchez-de-la-Torre et al., 2023). Indeed, adherence is arguably the greatest limitation of CPAP therapy. Based on the above observations, one could speculate that, if all patients adhered fully to CPAP, their cardiovascular outcomes would improve significantly. However, despite over three decades of technological advancements, CPAP adherence rates remain disappointingly low (Rotenberg et al., 2016).

In this narrative review, we discuss the current challenges that may undermine the effectiveness of CPAP therapy, and provide an overview of how the scientific community has sought to address these issues, including recent advancements in PAP delivery algorithms. To prepare this review, we conducted a comprehensive search of major scientific databases (PubMed, Google Scholar, ClinicalTrials.org) from their inception to the present, aiming to outline the current limitations of CPAP therapy and highlight potential future improvements.

## 2 Main limitations of CPAP

**Figure 1:**
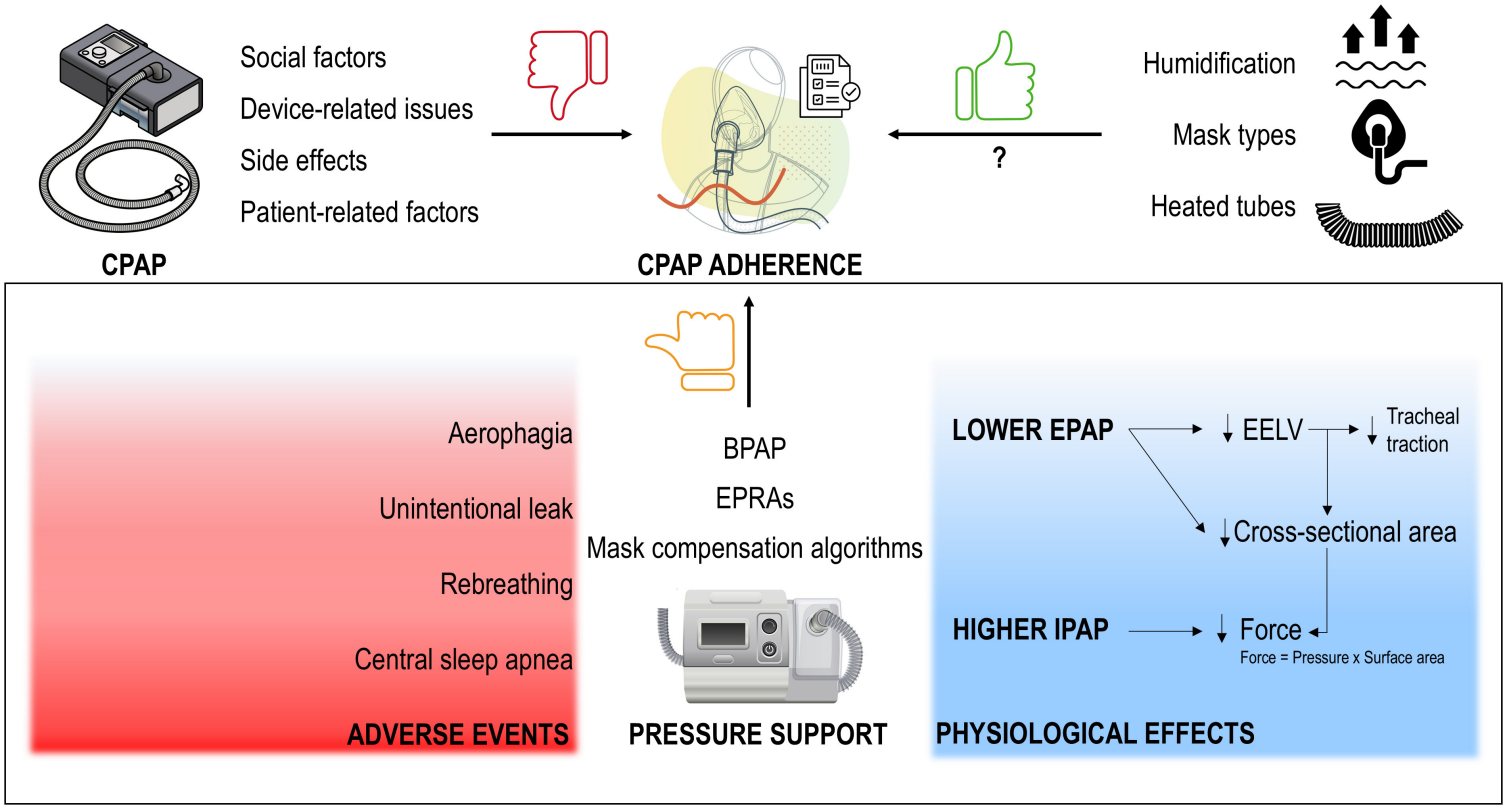
CPAP adherence is hindered by social factors, device- and patient-related issues and side effects **(top left)**. While technological advancements like heated humidifiers, heated tubes, and a wide variety of mask types **(top right)** may have enhanced the CPAP experience, data on adherence remain inconsistent, with adherence rates still low. Despite the early assumption that bilevel positive airway pressure (BPAP), with the introduction of pressure support [i.e., reducing expiratory PAP (EPAP) and potentially increasing inspiratory PAP (IPAP) for compensation], would improve adherence—based on the belief that higher pressures are required during inspiration—studies have shown this not to be the case. The ineffectiveness of expiratory pressure relief algorithms (EPRAs, such as C-Flex) and mask compensation algorithms, which mimic pressure support, further confirm this. Instead of improving adherence, these technologies often result in side effects **(bottom left)** and are less effective in maintaining upper airway patency, largely due to reductions in end-expiratory lung volume (EELV) and pharyngeal lumen cross-sectional area **(bottom right)**.

CPAP usage is hindered by social factors, device-related issues, side effects and patient-related factors (Figure 1). Socially, CPAP is often perceived as a burden by patients. Although the CPAP machine has been reduced by almost a third of its original size, it is still not easy to carry or transport conveniently, especially for frequent travel or daily mobility, and it may be cumbersome for patients to use outside of their home, limiting its practicality and ease of use. Another social factor affecting CPAP usage is inadequate support from bed partners, either due to non-dyadic sleep patterns (Lewis et al., 2004) or because the bed partner does not adequately encourage CPAP use or is disturbed by the noise from the device (Gentina et al., 2019).

A number of device-related factors can negatively impact the CPAP experience, such as mask discomfort, leaks, and noise from the device, although CPAP machines are much quieter now than in the past (Shirlaw et al., 2017; Pepin et al., 1995). In addition, CPAP can be responsible for circuit CO_2_ rebreathing (Messineo et al., 2024b). This may occur at low PAP levels or high respiratory volumes, when the patient exceeds the exhaust valve's flow capacity, and cause exhaled CO_2_ to flow back in the CPAP tubing to be inhaled during the next breath. The ensuing mild hypercapnia may cause anxiety, which, in turn, could lead to poor treatment adherence (Goossens et al., 2014; Woods et al., 1988).

Side effects impact up to 65% of users and include nasal congestion and epistaxis, xerostomia, facial lesions (i.e., skin marks or rashes), conjunctivitis, claustrophobia and aerophagy (Pepin et al., 1995; Brostrom et al., 2010; Strumpf et al., 1989). For many patients, exhaling against positive pressure can feel unnatural and may also contribute to insomnia (Ghadiri and Grunstein, 2020). These side effects are not always limited to the CPAP initiation. Although some tend to resolve with chronic usage, others can still emerge within the first year (Ulander et al., 2014). In addition, both social factors and side effects can exacerbate treatment-related anxiety over time and drive reduced adherence rates.

The propensity for low CPAP adherence can also be a consequence of factors inherently related to the patient, particularly abnormal OSA endotypes. As mentioned above, OSA can be the product of altered anatomy and a number of impaired physiological traits, such as high loop gain, low arousal threshold and poor pharyngeal muscle responsiveness (Messineo et al., 2024a,c). These traits can also contribute to reduced treatment adherence. For instance, patients with low arousal threshold, who tend to awaken easily in response to mild respiratory stimuli, may be more prone to frequent awakenings due to CPAP discomfort (e.g., noise, mask, etc.), leading to CPAP intolerance (Zinchuk et al., 2020). Additionally, patients with either very low or very high pharyngeal muscle responsiveness have shown lower adherence rates (Zinchuk et al., 2020). High loop gain has also been associated with improved adherence (Zinchuk et al., 2021), but a high loop gain is also linked the onset of central sleep apnea (CSA), which can negatively affect CPAP adherence (Cheng et al., 2024). A prospective study to assess the impact of physiological traits on PAP adherence is ongoing (Anwar et al., 2024).

At the end of the day, adherence is the ultimate problem. Patients experiencing CPAP-related social problems, device issues, or side effects may reduce their CPAP usage time or discontinue treatment altogether. Ultimately, how much can time on therapy be reduced and still be considered acceptable or effective? A threshold that has become clinically accepted worldwide is 4 h per night for at least 5 days a week (Kribbs et al., 1993). Also, many private insurers in the U.S. require this level of adherence for 30 days within the first 90 days of treatment to maintain CPAP coverage (Fujita et al., 2024). However, this threshold is arbitrary, and it is possible that, for example, only a full night of therapy 7 nights a week will improve OSA-related long-term outcomes. Nonetheless, this level of adherence seems out of reach for many patients. Indeed, early studies found adherence rates (per 4-h/night threshold) settling around 50% (Sawyer et al., 2011; Weaver and Grunstein, 2008). Two recent, real-world studies found adherence to be higher in the first 90 days of therapy (~75%) (Cistulli et al., 2019), as well as after 1 year (Morrone et al., 2020), with a mean CPAP usage time of ~5 h per night. However, this may still be inadequate, although comparable to adherence rates with pharmacotherapy for other chronic disease such as hypertension (Alsabbagh et al., 2014), diabetes (Cramer, 2004) and epilepsy (Getnet et al., 2016). In addition, adherence rates (Hwang et al., 2018) and usage time (Patel et al., 2022) tend to dramatically drop as early as 1 month after PAP prescription, and continue to decrease after 1 year of use. A recent parallel-group trial, assessed the effect of 6 months of a peer driven intervention (i.e., fully adherent CPAP patients acting as mentors for CPAP-naïve patients) on adherence in 263 participants. In the active control group, average adherence was < 4 h/night, with only 50% of patients reaching the recommendation of at least 4 h/night. Although the experimental intervention significantly increased CPAP adherence and was looked at as a therapeutic success, mean adherence went up by less than an hour, with only 62% of patients in the experimental treatment arm meeting the goal of 4 h of CPAP per night (Parthasarathy et al., 2024). Hence, these figures highlight that there is still abundant opportunity for further improvement in CPAP usage.

## 3 Technological improvements to address CPAP limitations

Over the past three decades, various efforts have been made to address the above CPAP limitations. Numerous examples of these technological innovations exist (Figure 1).

### 3.1 Humidification

Humidifiers were introduced in the ‘90s as a tool to reduce nasal symptoms (Parra et al., 1991). Without humidification, airflow through the nose dries the nasal mucosa and the faster the velocity of airflow, the more pronounced the drying will be (a phenomenon known as “jetting”). This leads to the release of vasoactive and proinflammatory mediators that increase superficial mucosal blood flow and cause vessel engorgement, particularly in the presence of mouth leaks, ultimately raising nasal resistance (Hayes et al., 1995). Hygroscopic humidifiers—or heated moisture exchangers—were shown to provide control of nasal symptoms and better adherence in early reports (Parra et al., 1991; Massie et al., 1999), although subsequent studies showed more mixed results. While some indicated improvement of nasal symptoms and inflammation following the use of heated humidifiers (Koutsourelakis et al., 2011; Rakotonanahary et al., 2001), others pointed at only modest-to-negligible improvement in CPAP adherence in the face of no benefits in daytime sleepiness or overall treatment satisfaction (Neill et al., 2003), especially in unselected OSA patients (Duong et al., 2005; Patil et al., 2019). Today, humidifiers are recommended only for patients experiencing nasal symptoms. Most CPAP devices now allow for customizable humidification levels based on individual needs.

### 3.2 Controlled heated breathing tube humidifiers

Introduced in the early 2000s, heated tubes represented another technological advancement aimed at preventing condensation buildup inside the tubing, especially at lower temperatures (Nilius et al., 2008), a common issue with earlier CPAP machines, that could disrupt airflow and comfort during use. Although heated tubes improved nasal symptoms for patients who prefer cooler bedroom environments (Nilius et al., 2008), they did not affect quality of life of OSA of patients or CPAP adherence in the short (Ruhle et al., 2011) or long term (Galetke et al., 2016) vs. conventional heated humidification.

### 3.3 Mask types

A wide variety of masks now provides patients with multiple options to achieve the best fit, enhancing comfort and minimizing leakage. In addition to selecting from full face masks, sub-nasal masks, nasal masks and nasal pillows, patients can also compare several mask brands. In addition, customizable 3D-printed masks have also been introduced to maximize fit (Hussin et al., 2021; Hsu et al., 2015). A well-fitting mask can help reduce pressure-related (e.g., skin lesions) and device-related (e.g., noise) side effects, depending on their geometry and intentional leak (Messineo et al., 2024b). However, the abundance of mask options may contribute to “choice fatigue” (Shah and Wolford, 2007), potentially leading to an incorrect initial selection and subsequent mask switching after therapy commencement, which can negatively impact CPAP adherence (Bachour et al., 2016). Although some differences in treatment adherence between nasal mask brands have been reported (Neuzeret and Morin, 2017), inconsistent findings indicate that, overall, there may not be a substantial difference in CPAP adherence between mask types (Patil et al., 2019; Benjafield et al., 2021; Rowland et al., 2018; Borel et al., 2013). Regardless, mask resupply has to be kept regularly executed to avoid sharp drops in adherence rates (Benjafield et al., 2021).

## 4 Pressure delivery algorithms to improve comfort

Despite the above improvements, which brought considerable reduction of nasal and facial symptoms, device-related issues (such as noise), and social factors (such as portability), adherence to CPAP therapy has not significantly improved, especially in the long run (Rotenberg et al., 2016). This suggests that the primary barrier to CPAP adherence may be intolerance to the pressure, particularly, it was believed, the discomfort experienced when exhaling against a positive pressure. Pressure intolerance may vary based on individual sensitivity, although some have hypothesized that persistent airway inflammation due to CPAP may be a contributor (Devouassoux et al., 2007).

Based on this notion, technology has evolved over the years to yield more tolerable methods of delivering pressure for CPAP therapy. However, almost all these advancements have not reached the goal of improving adherence (Figure 1), potentially due to negligible improvement in patient comfort related to PAP delivery.

### 4.1 Bilevel PAP (BPAP)

**Figure 2:**
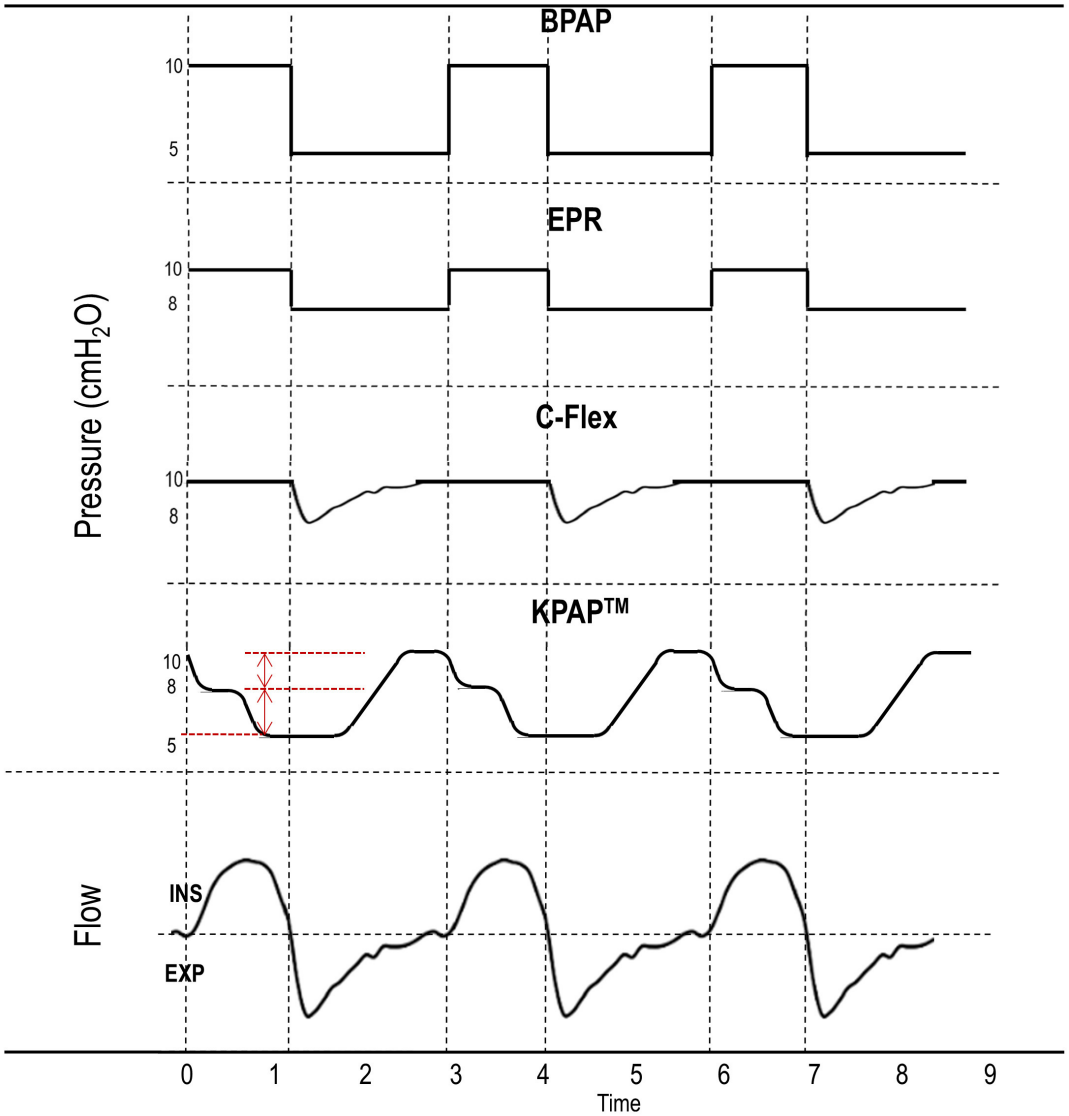
Different positive airway pressure (PAP) delivery algorithms. Bilevel PAP (BPAP) provides pressure support by delivering a higher inspiratory PAP (IPAP) than expiratory PAP (EPAP). Expiratory pressure relief (EPR) algorithms and C-Flex are features of continuous PAP devices designed to lower EPAP below therapeutic levels, thereby simulating pressure support (IPAP > EPAP). Kairos PAP™ (KPAP™) reduces pressure in two initial drops (red marks) starting in inspiration and returning to therapeutic levels only near the end of expiration (“the right time”). Inspiration (ins) is conventionally indicated by the flow curve above the dashed horizontal line, while expiration (exp) is the flow curve below the same line. Dashed vertical lines separate inspiration from expiration.

The introduction of BPAP in the early ‘90s was the first attempt to tackle pressure intolerance (Sanders and Kern, 1990). The prevailing view was that exhaling against a lower expiratory PAP (EPAP) could enhance comfort, while providing a higher inspiratory PAP (IPAP) could preserve upper airway patency. Specifically, it was thought that the normal negative inspiratory pressure which would tend to collapse the upper airway was not completely offset by pharyngeal muscle activation. Thus, airway narrowing forces were stronger during inspiration than exhalation (Remmers et al., 1978). To counteract these forces, higher pressures during inspiration were thought to be necessary, while reducing expiratory pressures was a logical predicate to reduce discomfort from exhaling against positive pressure. This approach was also believed to reduce mean pressure exposure while maintaining airway patency (Sanders and Kern, 1990). The notion that BPAP reduces the work of breathing by providing pressure support (IPAP > EPAP), where a higher IPAP supports inspiration and a lower EPAP reduces the effort needed for exhalation (Vitacca et al., 2001), was a further foundation for its use in OSA. A role for EPAP was still recognized, as it needed to be set above a critical level to prevent apneas and ensure sufficient airflow to trigger IPAP (Sanders and Kern, 1990; Resta et al., 1999). BPAP pressure curves are illustrated in Figure 2.

**Figure 3:**
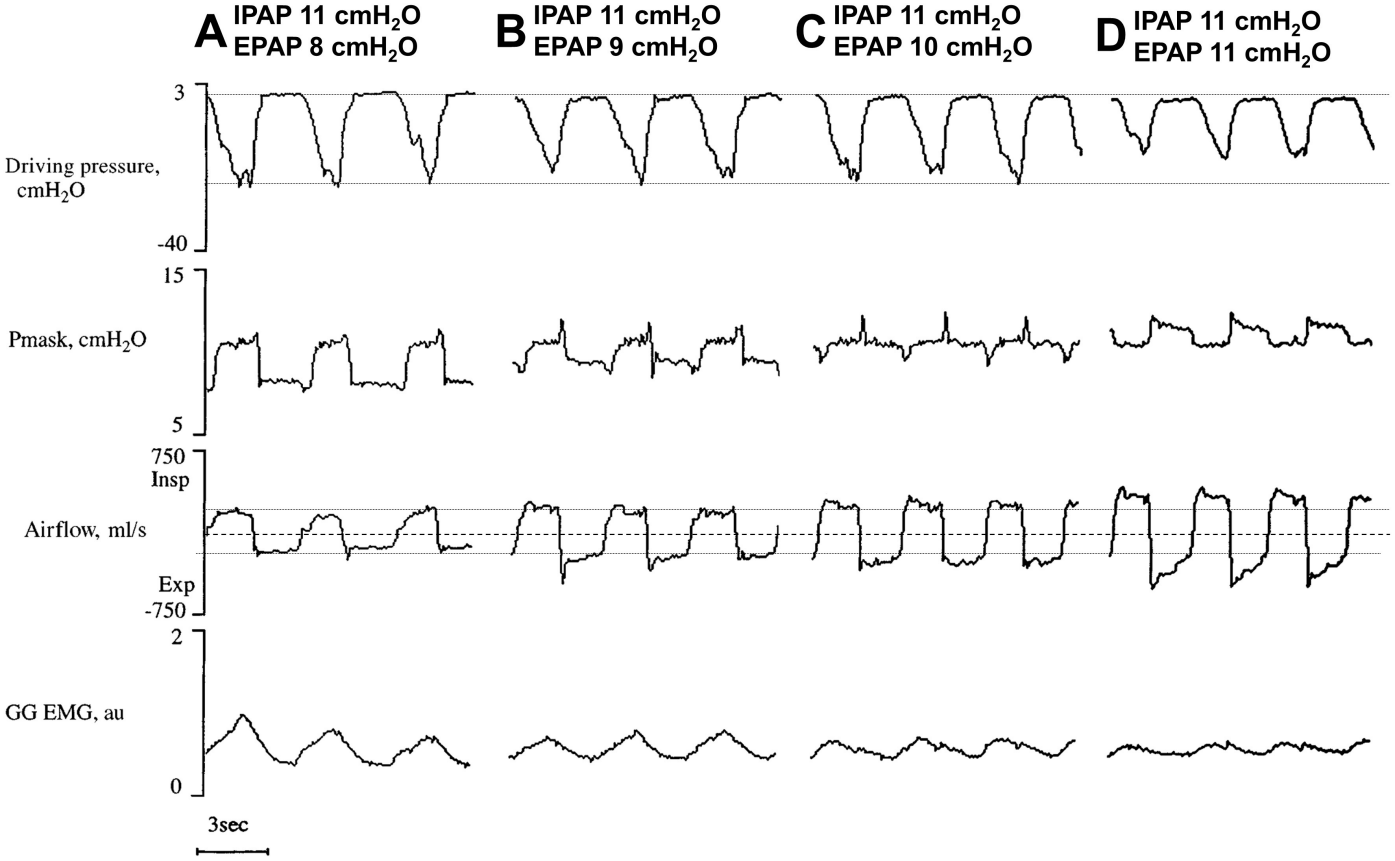
**(A–D)** Signal variations at increasing expiratory positive airway pressures (EPAP) with stable inspiratory PAP (IPAP). Note that as EPAP is reduced (reading right to left), peak flow steadily drops, almost certainly secondary to reduced airway patency. In addition, driving pressure [difference between mask pressure (Pmask) and esophageal pressure, representing efforts] and GG (genioglossus) electromyography (EMG) activity increase. Horizontal lines highlight zero flow (dashed) and swings in flow and driving pressure at minimal EPAP (dotted), to underscore increases in flow and decreases in effort with raising EPAP levels. Modified from Series and Marc (1998) (reproduced with permission).

However, multiple studies have shown that BPAP is not superior to CPAP in terms of adherence (Gay et al., 2003; Reeves-Hoche et al., 1995; Smith and Lasserson, 2009). It is possible that BPAP (or auto-adjusting BPAP) could be beneficial as a rescue therapy for selected OSA populations, such as patients specifically experiencing pressure intolerance (Benjafield et al., 2019; Carlucci et al., 2015; Palot et al., 2023), or obese individuals (Ishak et al., 2020); however, results on this topic have been inconsistent (Gulati et al., 2015). There is also evidence suggesting BPAP might actually destabilize the upper airway (Levy et al., 1994). Indeed, simply reducing EPAP leads to increased flow limitation (Figure 3) (Series and Marc, 1998), potentially necessitating an increase in IPAP beyond CPAP levels to offset this effect. This can lead to higher peak pressures and potentially increases the risk for central apnea events (see below).

### 4.2 Auto-titrating PAP (APAP)

APAP was initially introduced to eliminate in-laboratory titration, allowing the device to adjust pressure within a set range (minimum and maximum) over one night to a week, determining the pressure needed to treat respiratory events for 90% or 95% of the night (P90 and P95, respectively) (Rosen et al., 2012). However, APAP is now frequently used for routine treatment as it is commonly believed to improve adherence due to the reduction in overall pressure exposure. However, evidence supporting better adherence with APAP remains weak (Patil et al., 2019). In fact, fixed CPAP appears more effective on glycemic control (Shaw et al., 2016) and in lowering 24-h blood pressure (Pepin et al., 2016), and higher fixed pressures has been linked, in some studies, to better adherence (Van Ryswyk et al., 2019). Notably, both APAP and certainly BPAP have higher costs than CPAP.

### 4.3 Expiratory pressure relief algorithms (EPRAs)

Although most uncomplicated OSA patients do not routinely use BPAP at home, EPRA, such as C-Flex (proprietary to Philips Respironics) and EPR by ResMed, have become widely used in the past 20 years (Juhasz et al., 2001). EPRAs allow preferential reductions in EPAP (generally 1 to 3 cmH_2_O), thus providing low-level pressure support (i.e., IPAP > EPAP), in a similar fashion—though reduced in magnitude—to BPAP. The difference between various EPRAs, other than being proprietary to different manufacturers, lies within the shape of the curve of the reduced expiratory pressure (Figure 2). Each algorithm adjusts the pressure differently, affecting how smoothly or gradually the pressure is reduced during exhalation and returned prior to the next inspiration. Again, this technology was introduced based on the belief that IPAP was necessary to preserve upper airway patency and higher EPAP was simply uncomfortable. However, despite some pilot data showing a potential benefit of C-Flex with APAP vs. other EPRAs or vs. standard APAP (Chihara et al., 2013), overwhelming evidence indicates that neither C-Flex (Patil et al., 2019; Smith and Lasserson, 2009; Dolan et al., 2009; Bakker et al., 2010; Zhu et al., 2016), nor EPRAs (Patil et al., 2019; Smith and Lasserson, 2009), lead to improvements in adherence vs. standard CPAP, with the possible exception of C-Flex as a rescue therapy in patients with low CPAP tolerance (although long-term data supporting this finding are lacking) (Pepin et al., 2009a).

### 4.4 Mask compensation algorithms

Finally, CPAP manufacturers have introduced mask compensation algorithms, designed to offset the increased resistance associated with CPAP masks, particularly nasal pillows, which have the highest within-mask resistance. Since increased resistances are associated with drops in pressure, the algorithm compensates for such nasal-pillow-related drops in inspiratory pressure, providing a higher IPAP, and thus creating pressure support. However, there are no data to our knowledge supporting increased adherence with the use of this feature.

## 5 Potential reasons for why lower expiratory pressure does not increase adherence

There is ample evidence that pharyngeal collapse is not limited to inspiration—it also occurs during end-expiration (Sanders and Moore, 1983; Tamisier et al., 2004). The Starling resistor model, which represents the upper airway as a collapsible tube with intraluminal and surrounding pressures (Schwartz et al., 1988; Gold and Schwartz, 1996), supports the idea that complete airway obstruction primarily starts during expiration, when the airway muscles are most quiescent. In this model, during inspiration, when the surrounding pressure equals the downstream pressure (e.g., in the trachea), a choke point (i.e., flow limitation) ensues (Dawson and Elliott, 1980) and no further increase in airflow is possible, independent of further effort or further decrease in the downstream pressure. Therefore, according to this model, it is impossible to suck the airway fully closed. This means inspiratory negative pressure does not make the upper airway more susceptible to complete collapse (i.e., apneas), which is different from end-expiration. A study indicating minimal airway narrowing during inspiration and a reduction in pharyngeal size at end-expiration (when lung volume is at its nadir) further substantiated this model (Schwab et al., 1993). However, research has also shown that the upper airway exhibits a more complex, purely elastic behavior, where dynamic changes in the downstream pressure quickly affect the size of the airway (e.g., negative effort dependence) and there is potential for complete pharyngeal collapse also starting in inspiration (Owens et al., 2012). Therefore, this would not always reflect the dynamics of a Starling resistor and suggests that the upper airway could have a more complex behavior (Owens et al., 2012; Genta et al., 2016). The main neglected aspect in the purely elastic model is viscosity, which dictates that the airway will not return to its resting state immediately after a change in intraluminal pressure. Strong evidence to support this viscous nature is the ability of nasal EPAP (nEPAP) devices to treat hypopneas and snoring (Kryger et al., 2011), which can occur in inspiration. At the end of expiration with nEPAP, flow and pressure are both 0. Thus, any improvement on the subsequent inspiration (reduction or elimination of hypopneas and snoring) could be due to the viscous properties of the airway, which does not immediately collapse to its resting state.

Nonetheless, if airway collapse was typically assumed to occur during inspiration, lowering EPAP through BPAP or some EPRAs (i.e., especially those with a reduction in EPAP for the entire duration of expiration) would be at least as effective as CPAP in dilating the upper airway. However, this turned out not to be true (Figure 1). The upper airway is a very compliant structure whose area can increase substantially on CPAP (Schwab et al., 1996). Progressive decrements in EPAP are associated with dramatic declines in pharyngeal lumen cross-sectional area (Gugger and Vock, 1992) and reductions in airflow (Figure 3) (Series and Marc, 1998). Decreasing EPAP from therapeutic levels also causes a decrease in lung volume (Bishop et al., 1978). Drops in lung volumes can, in turn, negatively affect pharyngeal stiffness and cross-sectional area (Burger et al., 1992; Hoffstein et al., 1984), due to a loss of a tractional effect on the trachea and the upper airway (Owens et al., 2010; Kairaitis et al., 2007; Rowley et al., 1996). Tractional forces on the trachea refers to the mechanical pull on the upper airway that reduces compliance of the pharynx making it less collapsible. As lung volume decreases, this traction is diminished, reducing the pharyngeal cross-sectional area and making airway collapse more likely (Van de Graaff, 1988). Reductions in lung volume lead to sizable increases in CPAP requirements (Heinzer et al., 2005) and to worsening of OSA severity (Heinzer et al., 2006). Notably, a study showed that increasing EPAP does not necessarily translate into lung volume increments during sleep (Heinzer et al., 2008), possibly due to a servo mechanism that prevents hyperinflation. This suggests that some IPAP might be necessary to maintain adequate lung volumes in support of upper airway patency. However, significant lung volume reductions are observed at sleep onset (Hudgel and Devadatta, 1984; Stadler et al., 2010), or after shifting from lateral to supine position (Messineo et al., 2023). In otherwise healthy OSA patients, EPAP—and not IPAP—compensates for this drop in lung volume, addressing the ensuing worsening respiratory events.

**Figure 4:**
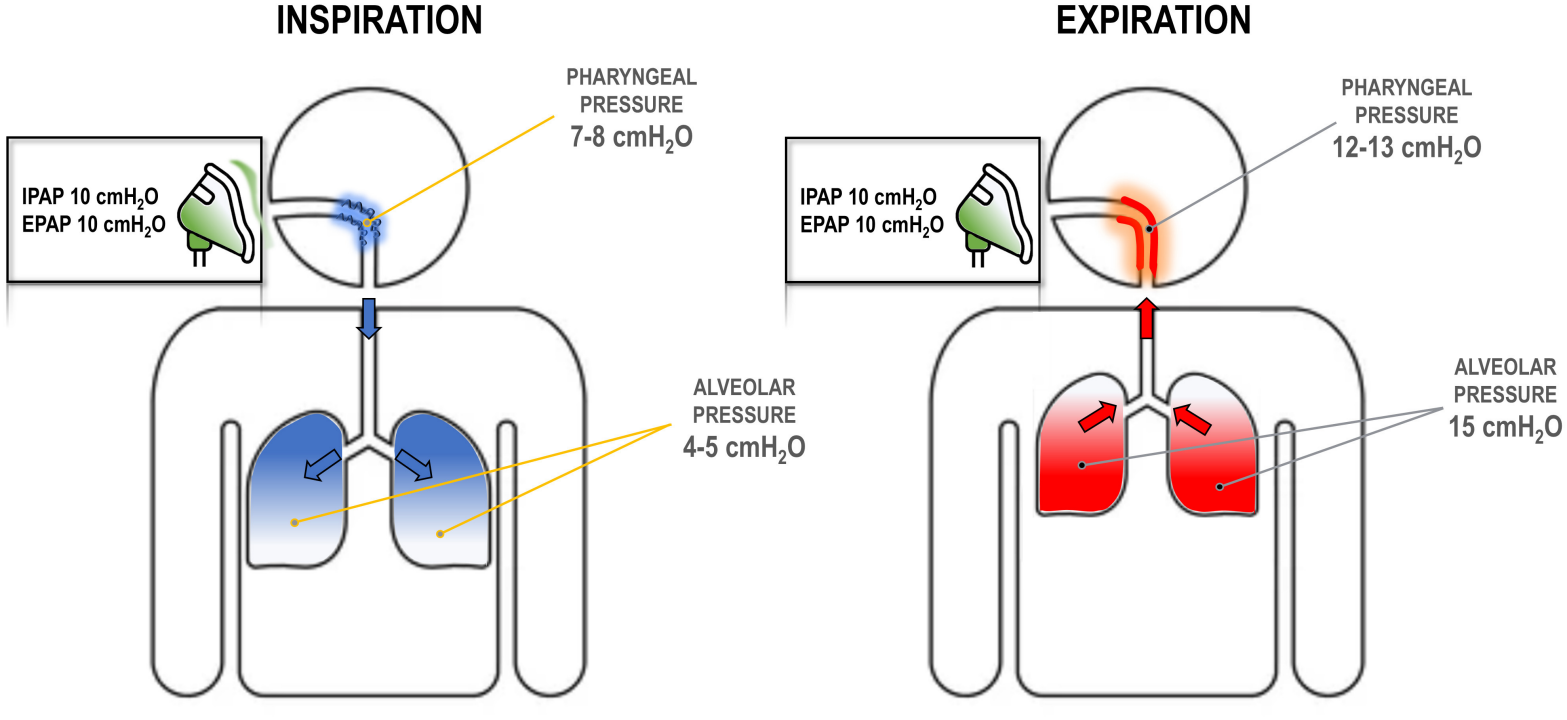
Respiratory dynamics at different positive airway pressure (PAP) settings. During inspiration **(left)**, the pressure at the mask dissipates as it travels to the pharynx due to high upper airway resistance and turbulent flow. For example, an inspiratory PAP (IPAP) of 10 cmH_2_O may reduce to 7–8 cmH_2_O, as some pressure is lost by overcoming the resistance. This pressure might be insufficient to keep the airway open. Alveolar pressure is lower as flow has to follow a pressure gradient. During expiration **(right)**, pressure in the alveoli must be higher than atmospheric pressure. Thus, with an expiratory PAP (EPAP) of 10 cmH_2_O, there must be sufficient pressure at the alveoli, e.g., 15 cmH_2_O, to create a pressure gradient. This pressure dissipates again in the pharynx due to resistance, but remains above the atmospheric pressure (e.g., 12–13 cmH_2_O) and contributes to stabilizing the upper airway.

On the other hand, higher IPAP does not dilate the upper airway for two key reasons. First, the set IPAP level is not fully delivered to the pharyngeal airway due to the resistance in the oronasal pathway, which dissipates the pressure (Figure 4). Second, since the pharyngeal cross-sectional area is smaller during inspiration (Schwab et al., 1993), there is less surface area for the positive pressure to act on. The force required to dilate the airway is the product of pressure and surface area, meaning that greater IPAP is needed to achieve the same upper airway dilation seen with EPAP (Figure 1). Therefore, reducing EPAP could lead to substantial increases in IPAP to maintain equivalent upper airway patency, or to increases in transthoracic pressure to preserve the same tidal volume (which could translate into an increased work of breathing). This should have been clear when Sanders et al. introduced bilevel pressure and noted that higher IPAP could not treat apneas, while lower EPAP could (Sanders and Kern, 1990).

Finally, it is possible that adding pressure support (IPAP > EPAP) increases the rate of unwanted central events (Figure 1), which can subsequently negatively affect adherence rates (Mulgrew et al., 2010). For example, augmented tidal volume from high IPAPs (Barach and Eckman, 1947; Campana et al., 2013) can trigger CSA (Zeineddine and Badr, 2021; Meza et al., 1998; Johnson and Johnson, 2005) or treatment emergent CSA (TECSA) (Malhotra et al., 2008), especially in individuals with high respiratory instability, i.e., high loop gain (Zeineddine and Badr, 2021). Even low levels of pressure support, such as with C-Flex, can elevate the likelihood of CSA (Loh et al., 2014) or TECSA (Noah et al., 2024). Several other adverse events linked to pressure support may contribute to reduced adherence. Decreasing EPAP heightens the chances of circuit-dependent CO_2_ rebreathing, especially with certain masks and at low PAPs. This occurs when the patient's exhaled airflow exceeds the exhaust flow (which is capped by mask specifications) and flows back into the CPAP tube. Thus, CO_2_ is rebreathed on the next inspiration (Messineo et al., 2024b). As aforementioned, rebreathing is a subtle cause of CPAP intolerance (Ferguson and Gilmartin, 1995).

High IPAP can also exacerbate unintentional leak, as leak is proportionate to the peak circuit pressure (Lebret et al., 2018). A pressure that is too high—such as with high IPAPs or with APAP devices which keep increasing EPAP to compensate for the leak—can displace the mask or be responsible for uncoupling of the tongue and soft palate, leading to mouth leak (Genta et al., 2020). Such unintentional leak has been described as a cause of decreased CPAP adherence (Rapoport, 1996; Baltzan et al., 2009). Indeed, leak can be disturbing to both the patient and the patient's bed-partner, and compromise their sleep architecture (Meyer et al., 1997).

Finally, high IPAP might induce more aerophagia by increasing the pressure gradient driving airflow into the esophagus. Aerophagia is a relatively common side effect of non-invasive ventilation (Gong and Sankari, 2024). Intuitively, increasing peak pressure as occurs with increased IPAP should raise the likelihood of aerophagia. This is supported by the observation that aerophagia is more prevalent with oronasal vs. nasal masks (Shirlaw et al., 2017), likely because oronasal masks have less resistance than nasal masks, leading to less pressure dissipation before the pharynx (i.e., IPAP at the pharynx is higher on oronasal vs. nasal masks).

**Figure 5:**
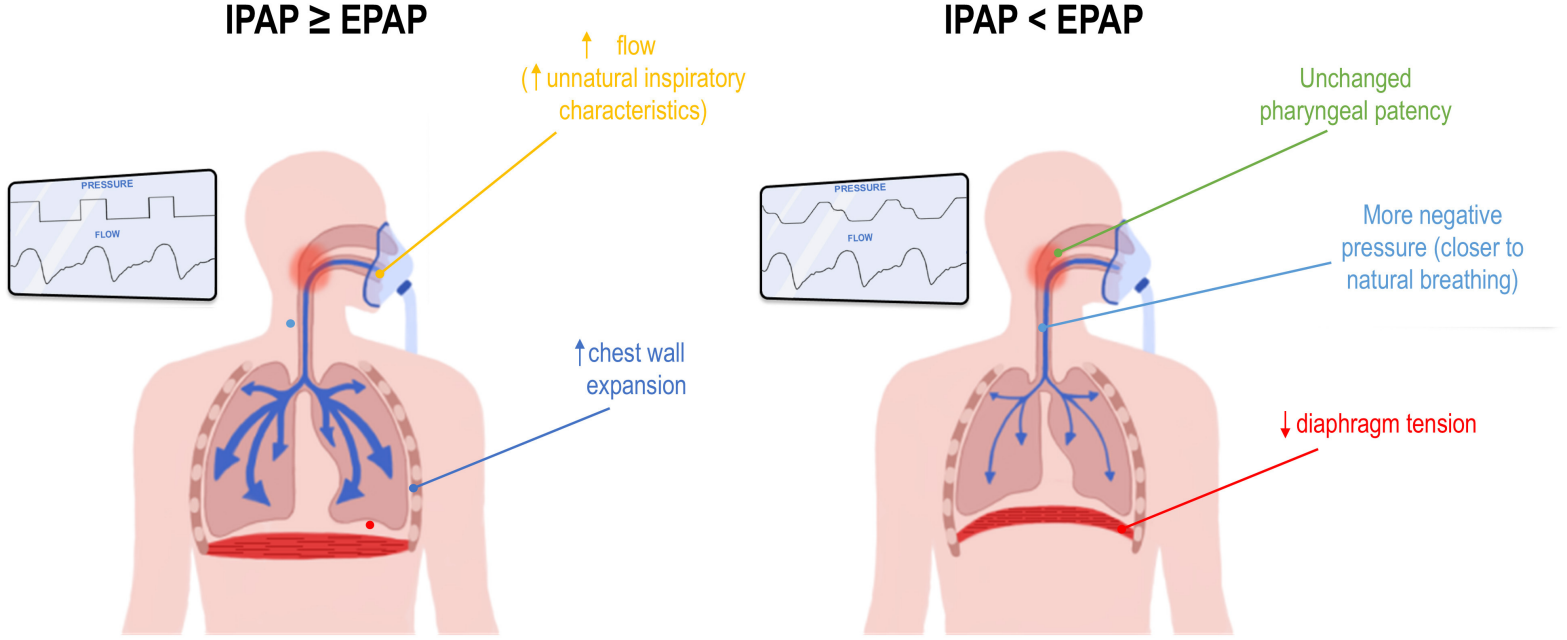
Potential mechanisms for improved comfort when inspiratory positive airway pressure (IPAP) is set lower than expiratory PAP (EPAP). This figure illustrates a hypothetical comparison between a device delivering IPAP equal to or higher than EPAP, as seen in continuous or bilevel PAP **(left inset)**, and a device delivering IPAP lower than EPAP, such as $\dot{\text{V}}$-Com^®^ or KairosPAP™ **(right inset)**. See text for full details.

It should also be noted that patients with uncomplicated OSA typically do not need pressure support to breathe, as their respiratory drive is intact and their lung physiology is quite normal. Since pressure support does not increase treatment adherence, the rationale behind administering IPAP > EPAP in otherwise healthy OSA patients seems misguided. Indeed, inspiring at higher pressures may be perceived as less comfortable, as the patient may feel less in control of their own inspiratory flow characteristics. They are thus “forced” into breaths by sudden pressure increases (Figure 5). Higher IPAP pressures also change the rate of chest wall expansion, diaphragm tension, and the flow rate through the pharynx when compared with natural breathing. Currently, the lowest IPAP that can be administered equals EPAP (i.e., CPAP). However, further lowering IPAP could more closely mimic natural breathing, namely breathing at lower pressures and at a pace that suits the individual patient. In addition, if pharyngeal narrowing primarily occurs at end-expiration, a lower IPAP should not compromise upper airway patency (Figure 5).

## 6 $\dot{\text{V}}$-Com^®^ as the first attempt to lower IPAP

$\dot{\text{V}}$-Com^®^ is a flow-dependent resistor, introduced in 2022 and FDA-approved as a class 1 device (similar to hoses, chinstraps, etc.), designed to enhance CPAP comfort by adding a non-compensated resistance to the CPAP circuit. Positioned in the tubing before the mask, $\dot{\text{V}}$-Com^®^ introduces a small amount of resistance that varies with flow, approximately 1.7 cmH_2_O at 50 L/min and increasing in a parabolic fashion—just over 0 cmH_2_O at 10 L/min and around 4.5 cmH_2_O at 70 L/min.

**Figure 6:**
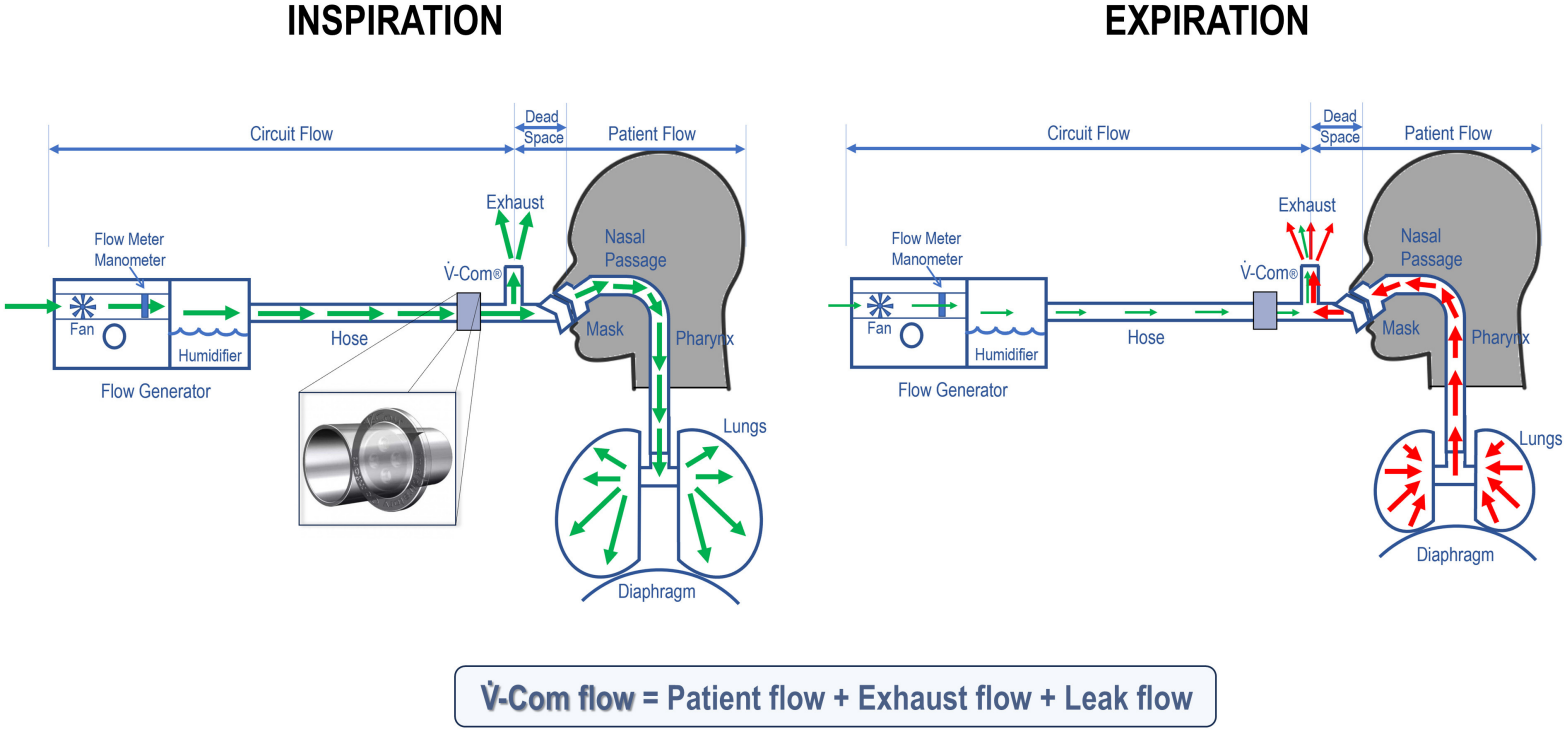
During inspiration **(left)**, the flow through $\dot{\text{V}}$-Com^®^ (inset) is high as it mainly comes from the flow that the patient generates to breathe air in (green arrows). As a result, there is a corresponding drop in IPAP when pressure goes through $\dot{\text{V}}$-Com^®^. During expiration **(right)**, the exhaled air (red arrows), in absence of rebreathing, exits through the exhaust valve and potentially the mouth (due to unintentional leak). Thus, the flow passing through $\dot{\text{V}}$-Com^®^ is minimal on expiration and there is no significant drop in EPAP.

As shown in Figure 6, when a patient is connected to a CPAP, the flow through $\dot{\text{V}}$-Com^®^ reflects the flow in the CPAP circuit. During inspiration, this is approximately 40–60 L/min, and accounts for both the machine's adjustments to maintain the set pressure and the patient's own inspiratory flow (5–8 L/min), plus the exhaust flow and potential leak flow. Importantly, the machine does not “breathe” for the patient, but supports the positive pressure required to keep the airway open and compensates for any leak in the system. Thus, the $\dot{\text{V}}$-Com^®^-related reduction in IPAP is approximately 1.5–2 cmH_2_O during inspiration. Conversely, during expiration, the machine adjusts flow minimally to maintain positive pressure, while most of the patient's exhaled air exits through the exhaust valve (unless in the presence of rebreathing), or through unintentional leak. Thus, during expiration, the flow going through $\dot{\text{V}}$-Com^®^ is minimal and there is negligible variation in EPAP.

In a study (Farney et al., 2024), 102 patients using an APAP device at home were studied for 4 days with $\dot{\text{V}}$-Com^®^ and 4 days without it, according to a crossover design. Patients were selected to be highly adherent to PAP therapy, with at least 6 documented hours of use per night over the previous 3 months. The patients were aware of the intervention. The first 62 patients were recruited independent of the CPAP device manufacturer they were using at home, while the last 40 were all using ResMed devices. Outcome data were downloaded from device built-in algorithms. In both patient groups, the introduction of $\dot{\text{V}}$-Com^®^ did not have a clinically meaningful effect on P90/P95, while it significantly decreased residual AHI vs. no $\dot{\text{V}}$-Com^®^ (1.79 ± 1.74 vs. 2.15 ± 2.35 events/hour, respectively, *P* = 0.017). This suggests that the reduced IPAP with $\dot{\text{V}}$-Com^®^ does not interfere with pressure delivery or the auto-titration algorithms, nor does it compromise pharyngeal patency. Rather, it highlights that expiration—as opposed to inspiration—is likely the most vulnerable phase for airway collapse, and maintaining therapeutic EPAP during at least a portion of the expiratory cycle is crucial in preventing respiratory events. Interestingly, in the first group, the $\dot{\text{V}}$-Com^®^ placement increased usage time by approximately 4% (from 7.3 ± 1.3 to 7.6 ± 1.4 h a night, *P* = 0.026), potentially secondary to reduced leakage (unintentional leak was significantly reduced by about a third in both groups—from 12.3 ± 9.3 to 8.1 ± 7.1 L/min, *P* < 0.001, in the first group, and from 8.8 ± 10.9 to 5.9 ± 6.4, *P* = 0.007, in the second group) and increased comfort. Indeed, 74% of all participants preferred to continue at-home therapy with $\dot{\text{V}}$-Com^®^ in place (however this was not further investigated in subsequent follow-ups). In summary, this study demonstrated that not only is IPAP < EPAP equally effective, but it may also be more comfortable for patients and pave the way for increased CPAP adherence.

In another study, the addition of $\dot{\text{V}}$-Com^®^ to the CPAP circuit during split-night titrations was able to resolve TECSA in 100% of the occurrences (Noah et al., 2024). In all 13 participants where TECSA was observed (~1% of the study cohort), overnight $\dot{\text{V}}$-Com^®^ placement systematically reduced the central apnea index from 17.3 ± 11.0 events/h (pre-$\dot{\text{V}}$-Com^®^ mean ± SD) to 1.5 ± 1.7 events/h. This effect was likely mediated by $\dot{\text{V}}$-Com^®^ reducing IPAP and consequently tidal volume (Bishop et al., 1978; Meza et al., 1998; Johnson and Johnson, 2005; Skatrud and Dempsey, 1983). Most likely, this helped stabilize breathing by driving end-tidal CO_2_ above the apnea threshold.

Thus, in conclusion, $\dot{\text{V}}$-Com^®^ maintained or improved therapy, and, unlike IPAP > EPAP, slightly enhanced adherence, reduced leak, and eliminated TECSA. However, it is important to note that $\dot{\text{V}}$-Com^®^ should be tested in a larger population and with a patient blinding intervention to ensure unbiased results. In addition, $\dot{\text{V}}$-Com^®^ was evaluated using a 6 cmH_2_O pressure range around the P90/P95 (minimum and maximum pressure were set 2 cmH_2_O below and 4 cmH_2_O above the P90/P95, respectively). Future studies should assess whether $\dot{\text{V}}$-Com^®^ affects OSA outcomes when wider PAP ranges are used and optimal P90/P95 is not already known, to confirm its usefulness in more real-world settings.

## 7 Evaluating lower inspiratory and expiratory pressures to improve pap adherence

KPAP™, derived from the Greek word Kairos, meaning “at the right time”, is a new pressure delivery algorithm providing full therapeutic pressure only at end-expiration, namely when the upper airway is more susceptible to narrowing or collapse. KPAP™, manufactured by SleepRes, takes further the concept of $\dot{\text{V}}$-Com^®^ by decreasing IPAP in the circuit to enhance comfort, featuring two sequential, flow-dependent reductions in pressure that begin at the initiation of inspiration and continue approximately halfway through expiration, before gradually returning to therapeutic pressure (Figure 2). The first pressure drop can be set at 1 or 2 cmH_2_O, while the second drop occurs at peak inspiratory flow, and can be adjusted to 1, 2, or 3 cmH_2_O. These combined reductions can reach a maximum total drop of 5 cmH_2_O, but IPAP (and part of EPAP) is never allowed to fall below 5 cmH_2_O. For example, if a patient's therapeutic pressure is set to 8 cmH_2_O, the maximum combined PAP drop would be 3 cmH_2_O (e.g., 2 + 1 cmH_2_O); similarly, if the baseline pressure is 9 cmH_2_O, the maximum PAP drop would be 4 cmH_2_O (i.e., 2 + 2 cmH_2_O), while it could be up to 5 cmH_2_O for baseline pressures of 10 cmH_2_O or above. The threshold of a 5 cmH_2_O maximum total drop was established based on preliminary observations from a local cohort of 150 patients, where comfort levels rose as IPAP was decreased, with subjective benefit increasing up to and plateauing at ~5 cmH_2_O. There was also concern that in some patients, pressure drops larger than 5 cmH_2_O might increase the frequency of disordered breathing events.

**Figure 7:**
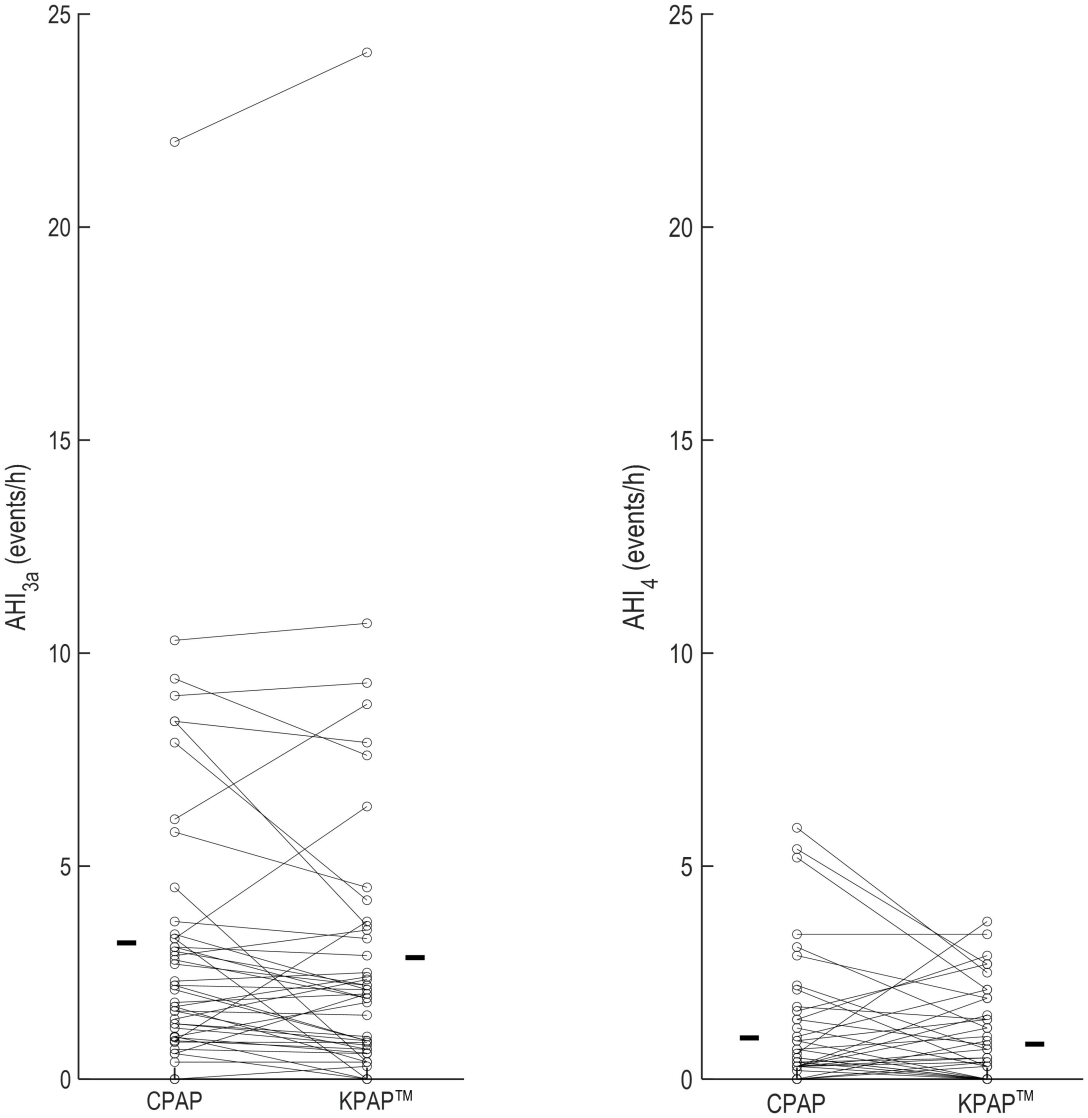
OSA severity metrics in a trial examining the effect of Kairos positive airway pressure (KPAP™) vs. continuous PAP (CPAP) in 48 participants (individual data are illustrated in figure) (White et al., 2024). AHI_3a_ indicates the apnea and hypopnea index when hypopneas were scored as a ≥30% reduction in flow plus a 3% oxyhemoglobin desaturation and/or the presence of a respiratory-related arousal. AHI_4_ was the AHI with hypopneas associated with 4% desaturations. In this trial, the effect of CPAP vs. KPAP™ on AHI_3a_ was the primary outcome, whereas AHI_4_ was assessed as part of sensitivity analyses. The bars in each panel are the mean values on the corresponding treatment arm. Reproduced with permission.

In a randomized, single-blinded, controlled trial, the effect of KPAP™ on OSA severity and patient's subjective comfort was investigated (White et al., 2024). KPAP™ was compared to CPAP in a randomized, crossover, split night design (half the night on KPAP™ and half on CPAP) to assess differences in residual AHI in 48 prospective participants with established OSA and evidence of at least 5 h of nightly APAP usage at home. Therapeutic CPAP pressure and baseline KPAP™ pressure were set to the P90/P95 determined by home APAP + 1 cmH_2_O. The study concluded that efficacy on residual events with KPAP™ was not inferior vs. with CPAP (mean difference [95%CI]; −0.6 [−10, −0.2] events/h in favor of KPAP™, *P* = 0.005; results were adjusted for supine sleep time, CPAP setting, mask type, period and sequence; Figure 7), confirming the findings observed with $\dot{\text{V}}$-Com^®^. Also similarly to $\dot{\text{V}}$-Com^®^, unintentional leak was reduced by roughly 40% on KPAP™ vs. CPAP.

**Figure 8:**
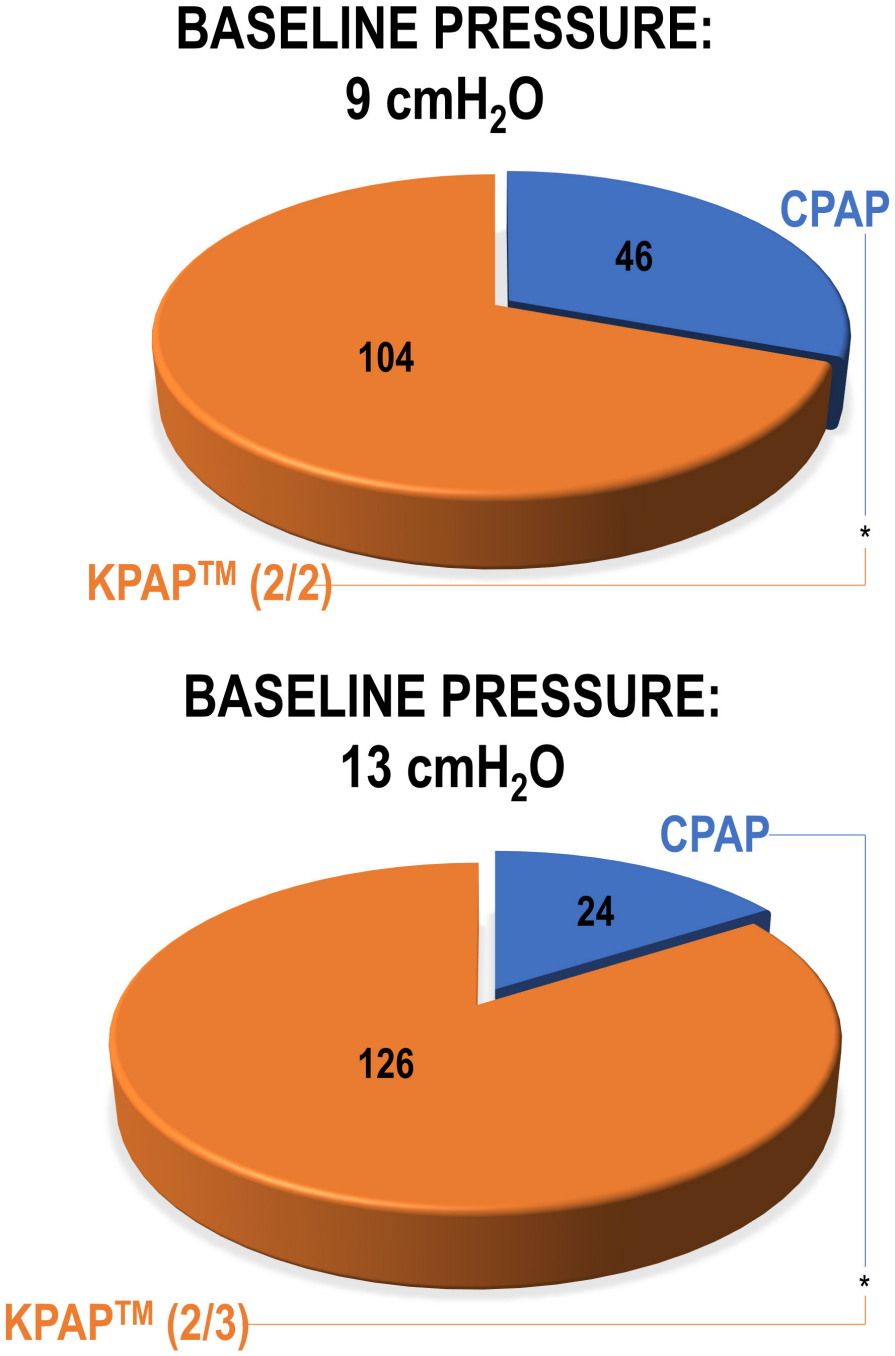
The pie charts illustrate participant preferences for Kairos positive airway pressure (KPAP™) vs. continuous PAP (CPAP) during a randomized office procedure conducted during wakefulness (White et al., 2024). Both KPAP™ and CPAP were administered at the baseline pressures of 9 cmH_2_O (first) and 13 cmH_2_O (later). In the KPAP arm, when the baseline pressure was 9 cmH_2_O, there was a 2 cmH_2_O drop at the start of inspiration and a subsequent drop of 2 cmH_2_O at peak inspiration [referred to as KPAP™ (2/2) in figure]. Similarly, at the baseline pressure of 13 cmH_2_O, pressure reductions were 2 and 3 cmH_2_O [KPAP™ (2/3) in figure]. Participants laid supine during PAP administration and raised their hand to indicate whether they preferred KPAP™ or CPAP. Results indicated a significant preference for KPAP™ at both baseline pressures (statistical significance illustrated by asterisks between treatments). Reproduced with permission.

To assess differences in comfort, CPAP at baseline pressures of 9 and 13 cmH_2_O vs. KPAP™ at the same therapeutic pressures with various drops—tailored to the baseline pressure—were administered in random order to 150 newly-diagnosed with OSA, CPAP-naïve participants (White et al., 2024). The study was carried out during wakefulness, during an in-office visit. After CPAP and KPAP™ administration, participants were asked to choose which treatment they found most comfortable. At a baseline pressure of 9 cmH_2_O, 69% of participants preferred KPAP™ (administered with pressure drops of 2 + 2 cmH_2_O; *P* < 0.001; Figure 8, top) over CPAP. At a baseline pressure of 13 cmH_2_O, 84% of participants preferred KPAP™ (administered with pressure drops of 2+3 cmH_2_O; P < 0.001; Figure 8, bottom). The study also assessed how many of the patients who initially preferred CPAP in the first round of choices found lower KPAP™ drops more comfortable. Eventually, overall, 93% of participants at 9 cmH_2_O and 95% at 13 cmH_2_O chose KPAP™ with one of the pressure drops over CPAP. This study had many limitations, including the absence of a wash-out period between treatments, or between home CPAP and the study night, and the sole focus on acute data. In practice, KPAP™ treatment could have benefitted from a residual effect of previous CPAP use (carryover effect) (Vroegop et al., 2015). However, no independent effect of sequence or period was observed, and AHI was systematically lower on KPAP™ than CPAP, indicating a low likelihood of a carryover effect.

In summary, KPAP™ confirmed and extended the preliminary findings observed with $\dot{\text{V}}$-Com^®^, including: (1) reducing IPAP by up to 5 cmH_2_O does not compromise upper airway patency, (2) targeting end-expiration as “the right time” to address pharyngeal narrowing appears to be an effective strategy, and (3) a lower IPAP is perceived as more comfortable, possibly due to a more natural breathing sensation (Figure 5). In addition, in consideration of the shared mechanism of action, it is possible that KPAP™ could have the same effect as $\dot{\text{V}}$-Com^®^ in solving TECSA. However, this will need to be confirmed in appropriate trials.

In addition, these findings will need to be confirmed in a larger cohort, with proper washout periods, and over longer treatment durations. Unselected OSA populations (i.e., not limited to individuals with prior good PAP adherence) will also need to be studied to evaluate the effect of IPAP-lowering interventions in those with known PAP intolerance or with factors that may affect it, such as specific endotypes like low arousal threshold. Finally, adherence data during long-term administration will also need to be collected.

Of note, since the impact of pressure on alveolar cells has been identified as a potential mechanism of increased systemic inflammation and adverse cardiovascular outcomes (Peker et al., 2024; Gottlieb et al., 2022; Shah et al., 2023), evaluating the long-term cardiovascular effects of a treatment delivering lower inspiratory pressure to the lungs could be highly valuable. Such an approach might not only reduce the risk of pressure-induced inflammatory responses but also provide new insights into optimizing PAP therapy to better prevent chronic adverse outcomes. Regardless, confirming that KPAP™ has at least the same effect of CPAP in reducing systemic blood pressure (Gottlieb et al., 2014; Messineo et al., 2024) and sleepiness (Pepin et al., 2009b; Weaver et al., 2007) would be valuable.

## 8 Conclusions

While CPAP has remained the cornerstone of treatment for OSA for over four decades, adherence continues to be a significant barrier to its long-term success. Despite technological improvements such as more comfortable mask designs, humidifiers, and pressure-relief algorithms, adherence rates remain suboptimal. Social factors, altered OSA endotypes, side effects and perception of lack of a therapeutic effect are some of the persistent challenges that ultimately lead to limited CPAP effectiveness.

Emerging solutions such as $\dot{\text{V}}$-Com^®^ and KairosPAP™, which offers a novel approach by reducing pressure at key points in the breathing cycle, demonstrate promising improvements in patient comfort without sacrificing efficacy. As these preliminary findings suggest, addressing specific comfort issues such as pressure intolerance may enhance long-term adherence. However, more research is necessary to assess the broader impact of KPAP™ and other innovations on patient outcomes and adherence over time. These results emphasize the need for continued advancements in PAP therapy to overcome the longstanding obstacles to patient compliance with the goal of improved outcomes in patients with obstructive sleep apnea.

## Data Availability

The original contributions presented in the study are included in the article/supplementary material, further inquiries can be directed to the corresponding author.
